# The impact of COVID-19 on screening for colorectal, gastric, breast, and cervical cancer in Korea

**DOI:** 10.4178/epih.e2022053

**Published:** 2022-06-21

**Authors:** Hyeree Park, Seung Hee Seo, Jong Heon Park, Shin Hye Yoo, Bhumsuk Keam, Aesun Shin

**Affiliations:** 1 Department of Preventive Medicine, Seoul National University College of Medicine, Seoul, Korea; 2 Cancer Research Institute, Seoul National University, Seoul, Korea; 3 Interdisciplinary Program in Cancer Biology Major, Seoul National University College of Medicine, Seoul, Korea; 4 Integrated Major in Innovative Medical Science, Seoul National University Graduate School, Seoul, Korea; 5 Big Data Steering Department, National Health Insurance Service, Wonju, Korea; 6 Center for Palliative Care and Clinical Ethics, Seoul National University Hospital, Seoul, Korea; 7 Department of Internal Medicine, Seoul National University Hospital, Seoul, Korea

**Keywords:** Colorectal neoplasms, Gastric neoplasms, Breast neoplasms, Uterine cervical neoplasms, Mass screening, COVID-19

## Abstract

**OBJECTIVES:**

The coronavirus disease 2019 (COVID-19) pandemic has affected the utilization of healthcare services, including participation in cancer screening programs. We compared cancer screening participation rates for colorectal, gastric, breast, and cervical cancers among participants in the National Cancer Screening Program (NCSP) in 2019 and 2020 to address the potential distraction effect of COVID-19 on cancer screening.

**METHODS:**

Data from the NCSP for 4 cancer types (stomach, colorectal, breast, and cervical) in 2019 and 2020 were used to calculate cancer screening participation rates by calendar month, gender, age group, and geographical region. Monthly participation rates were analyzed per 1,000 eligible individuals.

**RESULTS:**

The screening participation rate decreased in 2020 compared to 2019 for all 4 cancers: colorectal (40.5 vs. 35.3%), gastric (61.9 vs. 54.6%), breast (63.8 vs. 55.8%), and cervical (57.8 vs. 52.2%) cancers. Following 2 major COVID-19 waves in March and December 2020, the participation rates in the 4 types of cancer screening dropped compared with those in 2019. The highest decline was observed in the elderly population aged 80 years and older (percentage change: -21% for colorectal cancer; -20% for gastric cancer; -26% for breast cancer; -20% for cervical cancer).

**CONCLUSIONS:**

After the 2 major COVID-19 waves, the screening participation rate for 4 types of cancer declined compared with 2019. Further studies are needed to identify the indirect effects of the COVID-19 pandemic on cancer patients, such as delayed diagnoses of cancer or excess cancer deaths.

## GRAPHICAL ABSTRACT

[Fig f3-epih-44-e2022053]

## INTRODUCTION

After the first case of coronavirus disease 2019 (COVID-19) infection was reported in Wuhan, China, in December 2019, the World Health Organization declared COVID-19 a global pandemic in March 2020 [1,2]. The first case of a COVID-19-infected patient in Korea was reported in February 2020 [3]. The Korean government subsequently implemented social distancing regulations to prevent the spread of COVID-19. In Korea, social distancing guidelines have changed based on alert levels, which have been used to indicate the severity of the COVID-19 pandemic. For instance, in January 2020, with an alert level of yellow, major tertiary hospitals limited inpatient visits and installed thermal sensors at the hospitals’ entrances to detect COVID-19-infected patients with high fever [4]. Therefore, patients who were not infected with COVID-19 faced difficulties in accessing medical facilities, such as waiting for admission due to a shortage of hospital beds [5]. This situation can be considered a distraction effect, which refers to disruptions to healthcare systems or problems caused by other diseases [6]. The distraction effect due to COVID-19 might have impacted cancer patients because during the COVID-19 pandemic, most healthcare facilities focused on treating COVID-19 patients or preventing the spread of COVID-19 [7]. These circumstances may be associated with declines in cancer screening rates [8,9].

A decrease in the cancer screening participation rate can lead to delayed cancer diagnoses, which could translate into stage migration to more advanced cancer at diagnosis and a poorer prognosis for cancer patients [10]. In Taiwan, the uptake rate of colorectal cancer screening, defined as the number of individuals who submitted fecal immunochemical test (FIT) kits divided by the number of individuals to whom FIT kits were given, substantially declined compared to the 3-year period before the pandemic (December 2019 to April 2020: 88.1%, December 2016 to March 2019: 91.2-92.7%, p for trend < 0.001) [11]. In the United States, screening for colorectal, breast, and prostate cancers declined sharply during the COVID-19 pandemic. The estimated cancer screening deficits associated with COVID-19 were as follows: breast cancer, 3.9 million; colorectal cancer, 3.8 million; and prostate cancer, 1.6 million in 2020 compared with 2019 [12]. In another study conducted in the United States, cancer screening declined after the national emergency declaration (breast cancer, 96%; colorectal cancer, 95%). Concerns have been raised about excess deaths due to delayed cancer diagnoses caused by halted cancer screening programs [13]. In Japan, it has been suggested that delayed cancer screening during the pandemic may lead to diagnoses of cancer at advanced stages [14]. The anticipated outcome of interruptions in cancer screening programs not only includes delayed diagnoses, but also a marked increase in the number of avoidable cancer deaths [9].

In Korea, the National Cancer Screening Program (NCSP) was launched in 1999 and designed to provide free screening services for stomach, breast, and cervical cancers for the Medical Aid beneficiaries. Since then, the NCSP has continued to expand its target populations and target cancers [15,16]. As complete national lockdown measures were not implemented in Korea, the NCSP continued operations during the 3 major COVID-19 waves in the country, allowing us to assess changes in health-seeking behavior.

In this study, we analyzed the monthly changes in cancer screening participation rates for colorectal, gastric, breast, and cervical cancers among NCSP participants from different geographic regions and by age groups in 2020 compared to 2019 to address the potential distraction effect of COVID-19 on cancer screening.

## MATERIALS AND METHODS

### Data source and study population

The Korean NCSP provides cancer screening for 4 cancer types in the general population (gastric, breast, cervical, and colorectal cancers) and 2 cancer types in the high-risk population (liver and lung cancer). The eligible population of the NCSP includes National Health Insurance beneficiaries and all recipients of the Medical Aid Program. The eligible population differs according to the recommended screening interval for the type of cancer; for instance, annual screening is recommended for colorectal cancer, whereas biennial screening is recommended for gastric, breast, and cervical cancers. The target population for colorectal cancer screening with annual FITs is adults aged 50 years and older. For gastric cancer, all Korean adults aged 40 years and older are recommended to undergo endoscopic gastroduodenoscopy (EGD) or upper gastrointestinal (UGI) series every other year [17]. The NCSP recommends biennial breast cancer screening with mammography for women aged 40 years and older and biennial cervical cancer screening with Pap smears for women aged 20 years and older [18]. From the National Health Insurance Service (NHIS) database, we obtained information regarding the number of eligible individuals in 2019 and 2020, NCSP participants from January 2019 to December 2020, and the additional participants from January 1 to June 30, 2021 who did not receive cancer screening in 2020. The collected data included the number of monthly participants and socio-demographic information of the participants, such as gender, age in 5-year intervals, and residential information for 17 cities and provinces.

### Data analysis

The participation rate was defined as the number of participants divided by the eligible population. We compared the monthly screening participation rates for colorectal, gastric, breast, and cervical cancers in 2020 and in 2019 and then described them as a percentage change. We compared the monthly participation rates for each type of cancer screening per 1,000 eligible individuals. The percentage point difference (%p) was calculated by subtracting the participation rate in 2020 from that of the reference period (2019). Participation rates were analyzed by gender, age group, geographical region, and calendar month. We grouped the participants into age groups of 10-year intervals starting from the eligible screening age. The 17 cities and provinces were grouped into 4 geographical regions as follows: the capital region (Seoul, Gyeonggi, Incheon), central region (Daejeon, Sejong, Gangwon, Chungbuk, Chungnam), southwestern region (Gwangju, Jeonbuk, Jeonnam, Jeju), and southeastern region (Busan, Daegu, Ulsan, Gyeongbuk, Gyeongnam).

All analyses were conducted using Microsoft Excel (Microsoft, Redmond, WA, USA) and SAS version 9.4 (SAS Institute Inc., Cary, NC, USA).

### Ethics statement

The study protocol was approved by the Institutional Review Board of Seoul National University College of Medicine/Seoul National University Hospital (approval No. 2010-002-1160).

## RESULTS

The cancer screening participation rates decreased in 2020 compared with 2019 for all 4 cancers: colorectal (40.5 vs. 35.3%), gastric (61.9 vs. 54.6%), breast (63.8 vs. 55.8%), and cervical cancers (57.8 vs. 52.2%). As colorectal cancer screening is recommended every year, more individuals were eligible for colorectal cancer screening than were eligible to be screened for other cancer types (Table 1 and Supplementary Materials 1-4).

**Figure 1.**
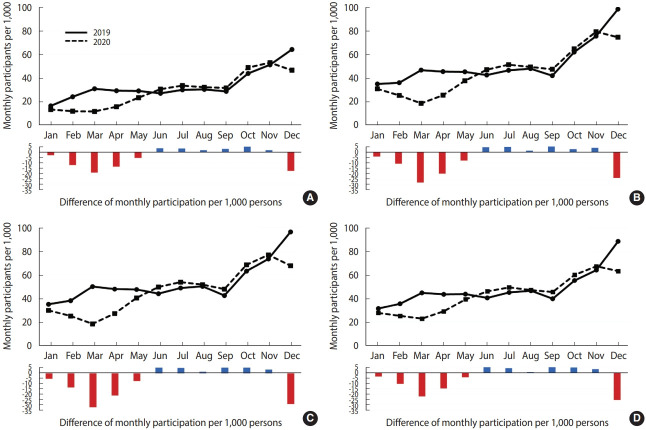
Monthly cancer screening participation rates and differences in monthly participants per 1,000 persons in 2020 compared with 2019. (A) Colorectal cancer, (B) gastric cancer, (C) breast cancer, (D) cervical cancer.

The screening participation rates for all 4 cancers declined in March and December 2020, which corresponded to the first and third COVID-19 waves, respectively. March 2020 had the sharpest drop in participation rates compared to March 2019 (percentage change: colorectal, -62.1%; gastric, -61.6%; breast, -63.1%; cervical, -49.2%). In June 2020, participation rates for the 4 cancer types increased compared to 2019 (percentage change: colorectal, +13.2%; gastric, +10.7%; breast, +13.1%; cervical, +14.1%), and then remained higher than those in the same months from 2019 until November 2020. By December 2020, during the third wave, the participation rates for screening had dropped compared with December 2019 (percentage change: colorectal, -27.4%; gastric, -24.3%; breast, -29.6%; cervical, -28.6%) (Figure 1).

We found a substantial decline in cancer screening participation rates in the elderly population, especially among those aged 80 years and older (Supplementary Materials 1-4). Colorectal and gastric cancer screening participation among people aged 80 years and older remained lower in 2020 than in 2019 (percentage change: colorectal, -21%; gastric, -20%) (Supplementary Materials 1 and 2). For breast cancer, the screening participation rate decreased in all age groups, and the largest decline was observed in women aged 80 years and older (percentage change: -26%) (Supplementary Material 3). Although the cervical cancer screening participation rate had been steadily increasing since 2010, it has decreased in all age groups in 2020 compared to 2019, with the exception of the 20-29 age group (Supplementary Material 4).

Table 1 shows the changes in cancer screening participation rates according to the geographical region. Considering the nationwide implementation of social distancing measures during each COVID-19 wave, we observed a similar pattern in cancer screening participation rates across Korea. For all 4 cancers, the capital region experienced the sharpest decline in 2020 compared with 2019 (highest percentage change: colorectal cancer, -13.0%); in contrast, the southwestern region experienced lower impacts for colorectal and cervical cancer (percentage change: colorectal, -11.9%; cervical, -8.7%).

## DISCUSSION

In this study, we observed a significant change in cancer screening participation rates in the Korean population during the COVID-19 pandemic in 2020. We analyzed the differences in detail according to calendar month, age group, gender, and geographical region. The monthly cancer participation rate decreased during the first COVID-19 wave in March 2020 and the third COVID-19 wave in December 2020. Similar patterns of monthly changes were observed in 17 cities and provinces in Korea. Cancer screening participation in the elderly population declined, especially in those aged 80 years and older.

**Figure 2.**
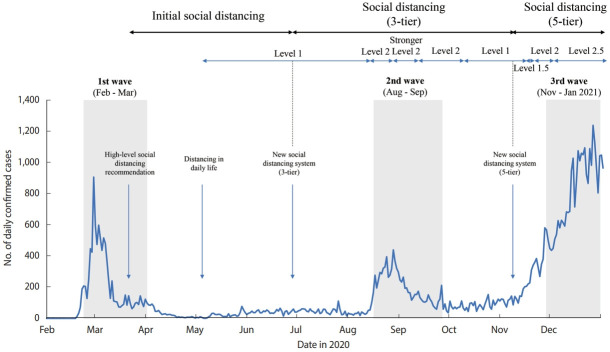
Changes in social distancing regulations from February to December 2020.

The participation rate in cancer screening decreased in March and December 2020 compared to the same months in 2019. During this period, the Korean government implemented strict social distancing regulations (Figure 2). The Korean government recommended high-level social distancing on March 22, 2020, and we found that the participation rate in cancer screening in March decreased compared to that in 2019 [19]. A similar pattern was observed in December 2020. After health authorities raised the social distancing level to level 2.5 in metropolitan areas on December 8, 2020, the second-highest in the new COVID-19 alert system, the cancer screening participation rate declined. Limited data are available to assess the continued impact of the COVID-19 pandemic on cancer screening and screening deficits in the long term [20]. In the United States, monthly changes in the cancer screening rate during the COVID-19 epidemic wave have been reported [12].

Difficulties in receiving proper care or participating in cancer screening during the COVID-19 pandemic have been identified in the elderly population of Ontario, Canada. The impact of the COVID-19 pandemic on cancer screening and diagnosis was estimated according to the change in the number of screening tests compared with 2019. Screening programs in Ontario delivered 41% fewer screening tests during the COVID-19 pandemic era than in 2019 [21]. Similar results were found in Korea, as the percentage change difference in the elderly population (over 80 years) was larger than that in the other age groups (percentage change: -21% for colorectal cancer; -20% for gastric cancer; -26% for breast cancer; -20% for cervical cancer). Another cross-sectional study reported the largest decrease in women aged 70 years and older (percentage change: -23%) among all age groups [22]. We could not find a definitive explanation for this decrease in cancer screening participation rates; however, a reason may be that older age can be a determinant in postponing cancer screening during the pandemic [23]. A study conducted in the United States suggested that social distancing is not the best measure to prevent the spread of COVID-19 in the elderly population, as it can cause increased morbidity and mortality due to a lack of social interaction and mental stimulation [24]. The reduction in cancer screening participation of the elderly population could also be attributed to hesitation to seek care due to fear of COVID-19 exposure in healthcare institutions [10].

Although cancer screening in Korea does not have an upper age limit, guidelines have been developed by expert committees and the National Cancer Center that recommend discontinuing cancer screening at the following ages for different cancers: colorectal cancer (80 years), gastric cancer (74 years), breast cancer (69 years), and cervical cancer (74 years) [25-28]. These guidelines are based on previous research on the prevalence, incidence, mortality, and cost-effectiveness of the early detection of cancer. According to a study based on a self-administered surveys of subjects who visited the health promotion center, 41.5% of health examinees preferred to continue receiving periodic health examinations with no upper age limit [29]. Our findings demonstrated a 14-18% decrease in the number of elderly participants who were above the recommended upper age limit in 2020 as compared with that of 2019. Since evidence indicates a small net benefit of screening all persons in the elderly population, participants and clinicians should consider individuals’ overall health and prior screening history in determining whether cancer screening is appropriate in specific cases [30]. Therefore, when interpreting the results, it should be kept in mind that a decrease in cancer screening participation of the elderly population does not always reflect negative health impacts from the public health perspective. Further studies should be conducted to gather evidence on the net benefit of cancer screening in adults who are above the upper age limit.

Breast and cervical cancer screening participation declined from 2019 to 2020. This finding is consistent with that of a study in a safety net hospital in San Francisco, where there was a reduction in the cumulative number of mammograms compared with the pre-COVID-19 era [22]. Another study reported that cervical cancer screenings declined after the national emergency declaration and remained 35% below the historical average [31]. The screening participation rate for colorectal and gastric cancer decreased more in women aged 70 years and older than in men of the same age group (percentage change: -12.4% in men vs. -16.6% in women for colorectal cancer; -12.0% in men vs. -14.7% in women for gastric cancer) (Supplementary Materials 1 and 2). Our data suggest that women were slightly more responsive to healthcare service utilization than were men.

Following the first identified case on January 20, 2020, the first wave of the COVID-19 pandemic in Korea began in February, and the first mass outbreak stemmed from a *Shincheonji* church in Daegu [32]. Subsequently, there was a decline in overall cancer screening participation in March 2020 compared to the same period in 2019 for all cancer types; a similar pattern was observed across different regions of the country (Supplementary Materials 5-8). The second COVID-19 wave occurred between August 13 and September 18, 2020, and many cases were identified in the densely populated Seoul metropolitan area [33]. The second wave ceased following stronger social distancing measures in the capital region during a 2-week period (August 30 to September 13). Unlike other countries where cancer screening rates dropped after national emergency declarations, the nationwide screening participation rates in the Korean population in September 2020 increased compared to those in the same month in 2019 for all 4 cancers (Supplementary Materials 5-8) [31]. In December 2020, at the time of the third wave, the screening participation rates for all cancer types decreased nationwide. Colorectal cancer screening rates decreased most sharply in the central region (percentage change: -2.6%), while the decline was largest for other cancer types in the capital region (percentage change: -2.7% for gastric cancer, -3.2% for breast cancer, -2.8% for cervical cancer).

The Korean government announced a 6-month extension of the NCSP for individuals who were eligible but had not participated in 2020 [34]. When the cancer screening participation rates of 2020 were recalculated with the inclusion of the individuals who participated during the 6-month extension period up to June 2021, the screening participation rates of 2020 were still lower than those of 2019 from the cancer types with biennial screening schedules (percentage change: -4.4% for gastric cancer; -5.6% for breast cancer; and -3.3% for cervical cancer) (Supplementary Material 9).

This study has several limitations. First, from our data, we could not analyze at the individual level whether the participants had postponed cancer screening to the following year. Considering that the 6-month extension of the NCSP notice was announced in late November 2020 by the Ministry of Health and Welfare, our data are thought to be sufficient for assessing the overall trends in cancer screening participation rates during the COVID-19 pandemic [34]. Another limitation is the lack of socioeconomic status information for the analysis. Therefore, it was not possible to address the challenges related to health equity during the pandemic. According to a community health survey that examined self-reported participation using a structured questionnaire, lower socioeconomic status was associated with lower participation in gastric cancer screening [35]. We could not assess this association from our data; however, considering that Medical Aid Program recipients and NHIS beneficiaries within the lower 50% income bracket can receive cancer screening services free of charge, whereas the NHIS beneficiaries in the upper 50% income bracket are charged a co-pay of 10% of the total cost of the procedure, it is suggested that other factors, such as indifference or inconvenience, could be a reason for non-participation in the cancer screening program compared to socioeconomic status alone [17]. A study on the trends in socioeconomic inequalities in cancer screening services in Korea reported that inequalities and inequities in participation in gastric and colorectal cancer screening were weakened after the implementation of the NCSP; however, only reducing out-of-pocket expenses for cancer screening may not completely eliminate the inequality [36]. Previous studies have identified health system-related characteristics as causes of non-participation [37]. Another study reported that barriers to cancer screening included lack of time, lack of knowledge about cancer screening programs, physical disability or underlying disease, and logistic barriers [38]. Finally, the eligible population varies in terms of different cancer types and their screening intervals. In the NCSP, annual screening is recommended for colorectal cancer, while biennial screening is recommended for gastric, breast, and cervical cancers. The eligible population in 2020 marginally decreased compared to that of 2019, and the difference in the eligible population should be taken into consideration for further comparison with follow-up data.

The main strength of the present study is its large dataset. The data used in this study are based on the NHIS database, which contains complete data on the cancer screening participation rate of the Korean population, including 23 million people aged over 20 years. To the best of our knowledge, this is the first study to demonstrate the decline in cancer screening participation in Korea according to major COVID-19 waves, age groups, and geographic regions.

In conclusion, it was observed that after the 2 major COVID-19 waves, the participation rates for FITs, EGDs or UGI series, mammographies, and Pap smears declined compared to those in 2019. There was a substantial decline during the first and third waves of the pandemic in March and December. The elderly population showed the highest reduction in the screening participation rate. The pattern of screening participation rate changes was comparable according to geographic region and gender.

The decline in cancer screening participation rates during the 2 major COVID-19 waves can be a potential indicator of the distraction effect of COVID-19. Even though the NCSP continued to function in 2020, the pandemic appears to have influenced the general population’s health-seeking behaviors. While the first and third waves of the pandemic initially stemmed from a specific region in Korea, there was a nationwide decline in cancer screening participation in March and December 2020. Cancer patient care may have been disrupted secondarily to COVID-19 through delayed cancer diagnoses because of reductions in cancer screening. Although the number of newly diagnosed cancer patients in Korea rose from 2016 to 2019, a 3% decrease was observed in 2020 when compared to 2019 [39]. A recent study conducted in the United States reported a significant decrease in the number of patients undergoing cancer screening tests and the ensuing diagnoses of cancerous and precancerous lesions during the COVID-19 pandemic [40]. Our study could provide evidence for the need for an essential healthcare delivery system in case of an upcoming pandemic, especially a community-level cancer screening program.

In contrast to reports showing a deficit in cancer screening in other countries, our results suggest a substantial recovery in cancer screening rates in Korea. Further studies are needed to identify the indirect effects of the COVID-19 pandemic on cancer patients, such as delayed diagnoses of cancer or excess cancer deaths.

## Figures and Tables

**Figure f3-epih-44-e2022053:**
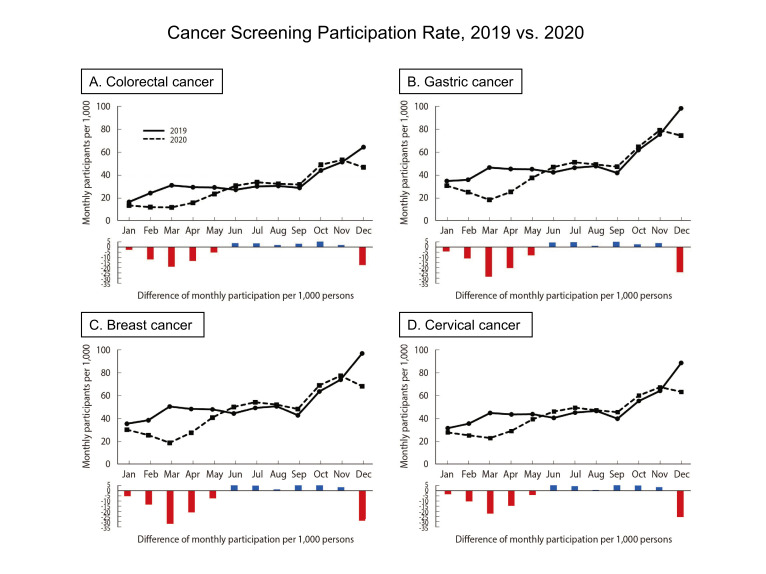


**Table 1.** Changes in cancer screening participation rates by geographical region, 2019 vs. 2020

Variables		Colorectal^1^	Gastric^2^	Breast^3^	Cervical^4^
Total					
2019	Eligible population	14,526,424	11,625,627	6,109,269	8,299,528
	Participants	5,886,319	7,194,489	3,894,928	4,799,842
	Participants per 1,000	405	619	638	578
	Participation rate (%)	40.5	61.9	63.8	57.8
2020	Eligible population	14,556,118	11,439,268	5,912,890	8,120,142
	Participants	5,135,163	6,244,491	3,299,516	4,240,658
	Participants per 1,000	353	546	558	522
	Participation rate (%)	35.3	54.6	55.8	52.2
	%p	-5.2	-7.3	-8.0	-5.6
	%	-12.8	-11.8	-12.5	-9.7
Capital					
2019	Eligible population	6,769,700	5,548,982	2,917,160	4,108,335
	Participants	2,830,807	3,372,486	1,859,299	2,411,769
	Participants per 1,000	418	608	637	587
	Participation rate (%)	41.8	60.8	63.7	58.7
2020	Eligible population	6,817,037	5,490,381	2,842,524	4,053,003
	Participants	2,479,458	2,923,408	1,579,110	2,139,826
	Participants per 1,000	364	532	556	528
	Participation rate (%)	36.4	53.2	55.6	52.8
	%p	-5.4	-7.5	-8.2	-5.9
	%	-13.0	-12.4	-12.8	-10.1
Central					
2019	Eligible population	1,122,616	1,635,797	845,308	1,122,616
	Participants	867,372	1,047,572	550,477	645,731
	Participants per 1,000	773	640	651	575
	Participation rate (%)	77.3	64.0	65.1	57.5
2020	Eligible population	1,094,473	1,600,580	818,088	1,094,473
	Participants	735,432	923,888	465,654	569,714
	Participants per 1,000	672	583	569	521
	Participation rate (%)	67.2	58.3	56.9	52.1
	%p	-10.1	-5.8	-8.2	-5.5
	%	-13.0	-9.0	-12.6	-9.5
Southwestern					
2019	Eligible population	1,771,950	1,378,301	722,990	930,931
	Participants	733,906	898,762	478,206	527,705
	Participants per 1,000	414	652	661	567
	Participation rate (%)	41.4	65.2	66.1	56.7
2020	Eligible population	1,750,459	1,336,719	692,605	905,455
	Participants	638,475	772,410	399,709	468,560
	Participants per 1,000	365	578	577	517
	Participation rate (%)	36.5	57.8	57.7	51.7
	%p	-4.9	-7.4	-8.4	-4.9
	%	-11.9	-11.4	-12.7	-8.7
Southeastern					
2019	Eligible population	3,894,743	3,062,547	1,623,811	2,137,646
	Participants	1,454,234	1,875,669	1,006,946	1,214,637
	Participants per 1,000	373	612	620	568
	Participation rate (%)	37.3	61.2	62.0	56.8
2020	Eligible population	3,921,727	3,011,588	1,559,673	2,067,211
	Participants	1,281,787	1,645,210	855,043	1,062,558
	Participants per 1,000	327	546	548	514
	Participation rate (%)	32.7	54.6	54.8	51.4
	%p	-4.7	-6.6	-7.2	-5.4
	%	-12.5	-10.8	-11.6	-9.5

1 Fecal immunochemical test.

2 Endoscopic gastroduodenoscopy or upper gastrointestinal series.

3 Mammography.

4 Pap smear.

## SUPPLEMENTARY MATERIALS

Supplementary Material 1. Colorectal Cancer Screening Participation Rate Change (2019 vs. 2020, % change and % point difference) by Gender and Age Group

Supplementary Material 2. Gastric Cancer Screening Participation Rate Change (2019 vs. 2020, % change and % point difference) by Gender and Age Group

Supplementary Material 3. Breast Cancer Screening Participation Rate Change (2019 vs. 2020, % change and % point difference) by Age Group

Supplementary Material 4. Cervical Cancer Screening Participation Rate Change (2019 vs. 2020, % change and % point difference) by Age Group

Supplementary Material 5. Colorectal Cancer Screening Participation Rate Change (2019 vs. 2020, % change and % point difference) per Month by Geographical Region

Supplementary Material 6. Gastric Cancer Screening Participation Rate Change (2019 vs. 2020, % change and % point difference) per Month by Geographical Region

Supplementary Material 7. Breast Cancer Screening Participation Rate Change (2019 vs. 2020, % change and % point difference) per Month by Geographical Region

Supplementary Material 8. Cervical Cancer Screening Participation Rate Change (2019 vs. 2020, % change and % point difference) per Month by Geographical Region

Supplementary Material 9. Cancer Screening Participation Rate in 2019 and 2020 considering the 6-month extension of the NCSP for the eligible population of 2020
