# Supplementary material for: The impact of COVID-19 on screening for colorectal, gastric, breast, and cervical cancer in Korea
Source: Epidemiol Health. 2022 Jun 21;44:e2022053. doi: 10.4178/epih.e2022053 (PMC9754922; doi:10.4178/epih.e2022053)
Supplement: Supplementary Material 1. — Colorectal Cancer Screening Participation Rate Change (2019 vs. 2020, % change and % point difference) by Gender and Age Group [file epih-44-e2022053-suppl1.docx]

# Supplementary Material 1. Colorectal Cancer Screening Participation Rate Change (2019 vs. 2020, % change and % point difference) by Gender and Age Group

|  |  | Age Group | Total | 50-59 | 60-69 | 70-79 | over 80 |
| --- | --- | --- | --- | --- | --- | --- | --- |
| Male | 2019 | Eligible Population | 6,750,556 | 3,006,815 | 2,091,199 | 1,191,677 | 460,865 |
|  |  | Participants | 2,716,039 | 1,027,801 | 979,439 | 570,030 | 138,769 |
|  |  | Participation Rate (%) | 40.2 | 34.2 | 46.8 | 47.8 | 30.1 |
|  | 2020 | Eligible Population | 6,814,372 | 2,978,249 | 2,151,127 | 1,201,954 | 483,042 |
|  |  | Participants | 2,385,235 | 871,614 | 880,840 | 513,824 | 118,957 |
|  |  | Participation Rate (%) | 35.0 | 29.3 | 40.9 | 42.7 | 24.6 |
|  | Difference | %p | 5.2 | -4.9 | -5.9 | -5.1 | -5.5 |
|  |  | % | -13 | -14 | -13 | -11 | -18 |
| Female | 2019 | Eligible Population | 7,775,868 | 3,039,526 | 2,365,270 | 1,502,634 | 868,438 |
|  |  | Participants | 3,172,964 | 1231378 | 1,149,682 | 629,380 | 162,524 |
|  |  | Participation Rate (%) | 40.8 | 40.5 | 48.6 | 41.9 | 18.7 |
|  | 2020 | Eligible Population | 7,741,744 | 2,932,260 | 2,428,610 | 1,489,893 | 890,981 |
|  |  | Participants | 2,749,927 | 1,036,527 | 1,049,967 | 536,926 | 126,507 |
|  |  | Participation Rate (%) | 35.5 | 35.3 | 43.2 | 36.0 | 14.2 |
|  | Difference | %p | -5.3 | -5.2 | -5.4 | -5.8 | -4.5 |
|  |  | % | -13 | -13 | -11 | -14 | -24 |
| Total | 2019 | Eligible Population | 14,526,424 | 6,046,341 | 4,456,469 | 2,694,311 | 1,329,303 |
|  |  | Participants | 5,886,319 | 2,256,495 | 2,129,121 | 1,199,410 | 301,293 |
|  |  | Participation Rate (%) | 40.5 | 37.3 | 47.8 | 44.5 | 22.7 |
|  | 2020 | Eligible Population | 14,556,117 | 5,910,510 | 4,579,737 | 2,691,847 | 1,374,023 |
|  |  | Participants | 5,135,162 | 1,908,141 | 1,930,807 | 1,050,750 | 245,464 |
|  |  | Participation Rate (%) | 35.3 | 32.3 | 42.2 | 39.0 | 17.9 |
|  | Difference | %p | -5.2 | -5 | -5.6 | -5.5 | -4.8 |
|  |  | % | -13 | -13 | -12 | -12 | -21 |
