# Supplementary material for: The impact of COVID-19 on screening for colorectal, gastric, breast, and cervical cancer in Korea
Source: Epidemiol Health. 2022 Jun 21;44:e2022053. doi: 10.4178/epih.e2022053 (PMC9754922; doi:10.4178/epih.e2022053)
Supplement: Supplementary Material 2. — Gastric Cancer Screening Participation Rate Change (2019 vs. 2020, % change and % point difference) by Gender and Age Group [file epih-44-e2022053-suppl2.docx]

Supplementary Material 2. Gastric Cancer Screening Participation Rate Change (2019 vs. 2020, % change and % point difference) by Gender and Age Group

|  |  | Age Group | Total | 40-49 | 50-59 | 60-69 | 70-79 | over 80 |  |  |
| --- | --- | --- | --- | --- | --- | --- | --- | --- | --- | --- |
| Male | 2019 | Eligible Population | 5,481,555 | 1,625,378 | 1,667,899 | 1,231,812 | 683,920 | 272,546 |  |  |
|  |  | Participants | 3,315,590 | 961,083 | 951,671 | 819,056 | 462,910 | 120,870 |  |  |
|  |  | Participation Rate (%) | 60.5 | 59.1 | 57.1 | 66.5 | 67.7 | 44.3 |  |  |
|  | 2020 | Eligible Population | 5,479,766 | 1,572,385 | 1,658,982 | 1,281,152 | 691,606 | 275,641 |  |  |
|  |  | Participants | 2,923,231 | 809,173 | 836,524 | 758,288 | 418,152 | 101,094 |  |  |
|  |  | Participation Rate (%) | 53.3 | 51.5 | 50.4 | 59.2 | 60.5 | 36.7 |  |  |
|  | Difference | %p | -7.2 | -7.7 | -6.6 | -7.3 | -7.2 | -7.7 |  |  |
|  |  | % | -12 | -13 | -12 | -11 | -11 | -17 |  |  |
| Female | 2019 | Eligible Population | 6,144,079 | 1,635,569 | 1,753,297 | 1,400,057 | 858,134 | 497,022 |  |  |
|  |  | Participants | 3,878,899 | 1,017,438 | 1,144,428 | 1,001,002 | 564,554 | 151,477 |  |  |
|  |  | Participation Rate (%) | 63.1 | 62.2 | 65.3 | 71.5 | 65.8 | 30.5 |  |  |
|  | 2020 | Eligible Population | 5,959,502 | 1,506,208 | 1,666,889 | 1,441,607 | 849,311 | 495,487 |  |  |
|  |  | Participants | 3,321,260 | 809,693 | 976,825 | 928,714 | 488,574 | 117,454 |  |  |
|  |  | Participation Rate (%) | 55.7 | 53.8 | 58.6 | 64.4 | 57.5 | 23.7 |  |  |
|  | Difference | %p | -7.4 | -8.4 | -6.7 | -7.1 | -8.3 | -6.8 |  |  |
|  |  | % | -12 | -14 | -10 | -10 | -13 | -22 |  |  |
| Total | 2019 | Eligible Population | 11,625,627 | 3,260,947 | 3,421,189 | 2,631,869 | 1,542,054 | 769,568 |  |  |
|  |  | Participants | 7,194,489 | 1,978,521 | 2,096,099 | 1,820,058 | 1,027,464 | 272,347 |  |  |
|  |  | Participation Rate (%) | 61.9 | 60.7 | 61.3 | 69.2 | 65.8 | 35.4 |  |  |
|  | 2020 | Eligible Population | 11,439,268 | 3,078,593 | 3,325,871 | 2,722,759 | 1,540,917 | 771,128 |  |  |
|  |  | Participants | 6,244,491 | 1,618,866 | 1,813,349 | 1,687,002 | 906,726 | 218,548 |  |  |
|  |  | Participation Rate (%) | 54.6 | 52.6 | 54.5 | 62.0 | 58.8 | 28.3 |  |  |
|  | Difference | %p | -7.3 | -8.1 | -6.7 | -7.1 | -7.8 | -7.0 |  |  |
|  |  | % | -12 | -13 | -11 | -10 | -12 | -20 |  |  |
