# Supplementary material for: The impact of COVID-19 on screening for colorectal, gastric, breast, and cervical cancer in Korea
Source: Epidemiol Health. 2022 Jun 21;44:e2022053. doi: 10.4178/epih.e2022053 (PMC9754922; doi:10.4178/epih.e2022053)
Supplement: Supplementary Material 3. — Breast Cancer Screening Participation Rate Change (2019 vs. 2020, % change and % point difference) by Age Group [file epih-44-e2022053-suppl3.docx]

Supplementary Material 3. Breast Cancer Screening Participation Rate Change (2019 vs. 2020, % change and % point difference) by Age Group

|  | Age Group | Total | 40-49 | 50-59 | 60-69 | 70-79 | over 80 |
| --- | --- | --- | --- | --- | --- | --- | --- |
| 2019 | Eligible Population | 6,109,269 | 1,636,676 | 1,745,101 | 1,391,240 | 844,969 | 491,283 |
|  | Participants | 3,894,928 | 1,064,206 | 1,174,298 | 1,002,225 | 525,314 | 128,885 |
|  | Participation Rate (%) | 63.8 | 65 | 67.3 | 72 | 62.2 | 26.2 |
| 2020 | Eligible Population | 5,912,890 | 1,504,174 | 1,655,649 | 1,428,065 | 835,480 | 489,522 |
|  | Participants | 3,299,516 | 845,088 | 996,993 | 916,185 | 445,850 | 95,400 |
|  | Participation Rate (%) | 55.8 | 56.2 | 60.2 | 64.2 | 53.4 | 19.5 |
| Difference | %p | -8.0 | -8.8 | -7.1 | -7.9 | -8.8 | -6.7 |
|  | % | -12 | -14 | -11 | -11 | -14 | -26 |
