# Supplementary material for: The impact of COVID-19 on screening for colorectal, gastric, breast, and cervical cancer in Korea
Source: Epidemiol Health. 2022 Jun 21;44:e2022053. doi: 10.4178/epih.e2022053 (PMC9754922; doi:10.4178/epih.e2022053)
Supplement: Supplementary Material 4. — Cervical Cancer Screening Participation Rate Change (2019 vs. 2020, % change and % point difference) by Age Group [file epih-44-e2022053-suppl4.docx]

Supplementary Material 4. Cervical Cancer Screening Participation Rate Change (2019 vs. 2020, % change and % point difference) by Age Group

|  | Age Group | Total | 20-29 | 30-39 | 40-49 | 50-59 | 60-69 | 70-79 | over 80 |
| --- | --- | --- | --- | --- | --- | --- | --- | --- | --- |
| 2019 | Eligible Population | 8,299,528 | 939,278 | 1,300,579 | 1,655,938 | 1,745,417 | 1,367,815 | 816,821 | 473,680 |
|  | Participants | 4,799,863 | 368,439 | 828,578 | 1,078,442 | 1,144,074 | 901,196 | 408,263 | 70,871 |
|  | Participation Rate (%) | 57.8 | 39.2 | 63.7 | 65.1 | 65.5 | 65.9 | 50 | 15 |
| 2020 | Eligible Population | 8,120,142 | 970,655 | 1,247,342 | 1,533,163 | 1,665,080 | 1,409,213 | 815,110 | 479,579 |
|  | Participants | 4,240,658 | 390,606 | 733,661 | 885,665 | 987,904 | 830,685 | 354,520 | 57,617 |
|  | Participation Rate (%) | 52.2 | 40.2 | 58.8 | 57.8 | 59.3 | 58.9 | 43.5 | 12 |
| Difference | %p | -5.6 | 1 | -4.9 | -7.4 | -6.2 | -6.9 | -6.5 | -2.9 |
|  | % | -10 | 3 | -8 | -11 | -9 | -11 | -13 | -20 |
