# Supplementary material for: The impact of COVID-19 on screening for colorectal, gastric, breast, and cervical cancer in Korea
Source: Epidemiol Health. 2022 Jun 21;44:e2022053. doi: 10.4178/epih.e2022053 (PMC9754922; doi:10.4178/epih.e2022053)
Supplement: Supplementary Material 5. — Colorectal Cancer Screening Participation Rate Change (2019 vs. 2020, % change and % point difference) per Month by Geographical Region [file epih-44-e2022053-suppl5.docx]

Supplementary Material 5. Colorectal Cancer Screening Participation Rate Change (2019 vs. 2020, % change and % point difference) per Month by Geographical Region

|  | | Eligible population |  | Total | Jan | Feb | Mar | Apr | May | Jun | Jul | Aug | Sep | Oct | Nov | Dec |
| --- | --- | --- | --- | --- | --- | --- | --- | --- | --- | --- | --- | --- | --- | --- | --- | --- |
| **Total** | |  |  |  |  |  |  |  |  |  |  |  |  |  |  |  |
| 2019 | | 14,526,424 | Participant | 5,886,319 | 238,524 | 350,333 | 448,163 | 425,007 | 421,487 | 392,814 | 436,659 | 441,415 | 417,161 | 638,038 | 743,595 | 933,123 |
|  |  |  | Participants per 1,000 | 405 | 16 | 24 | 31 | 29 | 29 | 27 | 30 | 30 | 29 | 44 | 51 | 64 |
| 2020 | | 14,556,118 | Participant | 5,135,163 | 195,904 | 172,884 | 170,023 | 228,138 | 340,204 | 445,566 | 489,150 | 468,005 | 461,219 | 711,916 | 773,085 | 679,069 |
|  |  |  | Participants per 1,000 | 353 | 13 | 12 | 12 | 16 | 23 | 31 | 34 | 32 | 32 | 49 | 53 | 47 |
|  | |  | %p | -5.2 | -0.3 | -1.2 | -1.9 | -1.4 | -0.6 | 0.4 | 0.4 | 0.2 | 0.3 | 0.5 | 0.2 | -1.8 |
|  | |  | % | -12.9 | -18 | -50.8 | -62.1 | -46.4 | -19.4 | 13.2 | 11.8 | 5.8 | 10.3 | 11.4 | 3.8 | -27.4 |
| **Capital** | |  |  |  |  |  |  |  |  |  |  |  |  |  |  |  |
| 2019 | | 6,769,700 | Participant | 2,830,807 | 84,729 | 151,606 | 202,938 | 201,050 | 205,588 | 196,892 | 210,369 | 215,576 | 205,330 | 316,572 | 370,085 | 470,072 |
|  |  |  | Participants per 1,000 | 418 | 13 | 22 | 30 | 30 | 30 | 29 | 31 | 32 | 30 | 47 | 55 | 69 |
| 2020 | | 6,817,037 | Participant | 2,479,458 | 72,661 | 69,368 | 84,293 | 109,237 | 162,085 | 206,400 | 233,245 | 227,684 | 227,514 | 355,844 | 390,112 | 341,015 |
|  |  |  | Participants per 1,000 | 364 | 11 | 10 | 12 | 16 | 24 | 30 | 34 | 33 | 33 | 52 | 57 | 50 |
|  | |  | %p | -5.4 | -0.2 | -1.2 | -1.8 | -1.4 | -0.7 | 0.1 | 0.3 | 0.2 | 0.3 | 0.5 | 0.3 | -1.9 |
|  | |  | % | -13.0 | -14.8 | -54.6 | -58.8 | -46.0 | -21.7 | 4.1 | 10.1 | 4.9 | 10.0 | 11.6 | 4.7 | -28.0 |
| **Central** | |  |  |  |  |  |  |  |  |  |  |  |  |  |  |  |
| 2019 | | 1,122,616 | Participant | 867,372 | 49,648 | 59,529 | 69,956 | 63,132 | 57,891 | 55,098 | 65,648 | 65,640 | 63,301 | 92,490 | 102,332 | 122,707 |
|  |  |  | Participants per 1,000 | 773 | 44 | 53 | 62 | 56 | 52 | 49 | 58 | 58 | 56 | 82 | 91 | 109 |
| 2020 | | 1,094,473 | Participant | 735,432 | 38,014 | 31,432 | 24,264 | 33,611 | 48,750 | 64,045 | 72,526 | 63,751 | 64,394 | 99,301 | 104,694 | 90,650 |
|  |  |  | Participants per 1,000 | 672 | 35 | 29 | 22 | 31 | 45 | 59 | 66 | 58 | 59 | 91 | 96 | 83 |
|  | |  | %p | -10.1 | -0.9 | -2.4 | -4.0 | -2.6 | -0.7 | 0.9 | 0.8 | 0.0 | 0.2 | 0.8 | 0.5 | -2.6 |
|  | |  | % | -13.0 | -21.5 | -45.8 | -64.4 | -45.4 | -13.6 | 19.2 | 13.3 | -0.4 | 4.3 | 10.1 | 4.9 | -24.2 |
| **Southwestern** | |  |  |  |  |  |  |  |  |  |  |  |  |  |  |  |
| 2019 | | 1,771,950 | Participant | 733,906 | 40,507 | 53,193 | 60,809 | 51,372 | 49,080 | 42,914 | 50,469 | 49,298 | 49,758 | 77,330 | 88,852 | 120,324 |
|  |  |  | Participants per 1,000 | 414 | 23 | 30 | 34 | 29 | 28 | 24 | 28 | 28 | 28 | 44 | 50 | 68 |
| 2020 | | 1,750,459 | Participant | 638,475 | 33,811 | 26,793 | 25,685 | 30,261 | 43,622 | 57,653 | 55,433 | 51,352 | 51,977 | 85,524 | 89,666 | 86,698 |
|  |  |  | Participants per 1,000 | 365 | 19 | 15 | 15 | 17 | 25 | 33 | 32 | 29 | 30 | 49 | 51 | 50 |
|  | |  | %p | -4.9 | -0.4 | -1.5 | -2.0 | -1.2 | -0.3 | 0.9 | 0.3 | 0.2 | 0.2 | 0.5 | 0.1 | -1.8 |
|  | |  | % | -11.9 | -15.5 | -49.0 | -57.2 | -40.4 | -10.0 | 36.0 | 11.2 | 5.4 | 5.7 | 12.0 | 2.2 | -27.1 |
| **Southeastern** | |  |  |  |  |  |  |  |  |  |  |  |  |  |  |  |
| 2019 | | 3,894,743 | Participant | 1,454,234 | 63,640 | 86,005 | 114,460 | 109,453 | 108,928 | 97,910 | 110,173 | 110,901 | 98,772 | 151,646 | 182,326 | 220,020 |
|  |  |  | Participants per 1,000 | 373 | 16 | 22 | 29 | 28 | 28 | 25 | 28 | 28 | 25 | 39 | 47 | 56 |
| 2020 | | 3,921,727 | Participant | 1,281,787 | 51,418 | 45,291 | 35,781 | 55,029 | 85,747 | 117,468 | 127,946 | 125,207 | 117,334 | 171,247 | 188,613 | 160,706 |
|  |  |  | Participants per 1,000 | 327 | 13 | 12 | 9 | 14 | 22 | 30 | 32 | 32 | 30 | 44 | 48 | 41 |
|  | |  | %p | -4.7 | -0.3 | -1.1 | -2.0 | -1.4 | -0.6 | 0.5 | 0.4 | 0.3 | 0.5 | 0.5 | 0.1 | -1.6 |
|  |  | | % | -12.5 | -19.8 | -47.7 | -69.0 | -50.1 | -21.8 | 19.1 | 15.3 | 12.1 | 18.0 | 12.1 | 2.7 | -27.5 |
