# Supplementary material for: The impact of COVID-19 on screening for colorectal, gastric, breast, and cervical cancer in Korea
Source: Epidemiol Health. 2022 Jun 21;44:e2022053. doi: 10.4178/epih.e2022053 (PMC9754922; doi:10.4178/epih.e2022053)
Supplement: Supplementary Material 6. — Gastric Cancer Screening Participation Rate Change (2019 vs. 2020, % change and % point difference) per Month by Geographical Region [file epih-44-e2022053-suppl6.docx]

Supplementary Material 6. Gastric Cancer Screening Participation Rate Change (2019 vs. 2020, % change and % point difference) per Month by Geographical Region

|  | Eligible population |  | Total | Jan | Feb | Mar | Apr | May | Jun | Jul | Aug | Sep | Oct | Nov | Dec |
| --- | --- | --- | --- | --- | --- | --- | --- | --- | --- | --- | --- | --- | --- | --- | --- |
| **Total** |  |  |  |  |  |  |  |  |  |  |  |  |  |  |  |
| 2019 | 11,625,627 | Participant | 7,194,489 | 400,488 | 413,433 | 537,228 | 522,844 | 521,641 | 488,272 | 536,443 | 552,960 | 482,632 | 717,136 | 876,125 | 1,145,287 |
|  |  | Participants per 1,000 | 619 | 34 | 36 | 46 | 45 | 45 | 42 | 46 | 48 | 42 | 62 | 75 | 99 |
| 2020 | 11,439,268 | Participant | 6,244,491 | 345,883 | 281,468 | 202,853 | 283,708 | 423,812 | 532,061 | 582,320 | 559,542 | 536,999 | 737,299 | 905,988 | 852,558 |
|  |  | Participants per 1,000 | 546 | 30 | 25 | 18 | 25 | 37 | 47 | 51 | 49 | 47 | 64 | 79 | 75 |
|  |  | %p | -7.3 | -0.4 | -1.1 | -2.8 | -2.0 | -0.8 | 0.5 | 0.5 | 0.1 | 0.5 | 0.3 | 0.4 | -2.4 |
|  |  | % | -11.8 | -12.2 | -30.8 | -61.6 | -44.9 | -17.4 | 10.7 | 10.3 | 2.8 | 13.1 | 4.5 | 5.1 | -24.3 |
| **Capital** |  |  |  |  |  |  |  |  |  |  |  |  |  |  |  |
| 2019 | 5,548,982 | Participant | 3,372,486 | 145,248 | 171,766 | 233,345 | 235,203 | 241,731 | 233,292 | 250,416 | 262,742 | 233,343 | 354,017 | 438,116 | 573,267 |
|  |  | Participants per 1,000 | 608 | 26 | 31 | 42 | 42 | 44 | 42 | 45 | 47 | 42 | 64 | 79 | 103 |
| 2020 | 5,490,381 | Participant | 2,923,408 | 129,028 | 110,937 | 92,162 | 125,276 | 190,070 | 237,999 | 269,219 | 267,919 | 261,747 | 364,365 | 453,560 | 421,126 |
|  |  | Participants per 1,000 | 532 | 24 | 20 | 17 | 23 | 35 | 43 | 49 | 49 | 48 | 66 | 83 | 77 |
|  |  | %p | -7.5 | -0.3 | -1.1 | -2.5 | -2.0 | -0.9 | 0.1 | 0.4 | 0.1 | 0.6 | 0.3 | 0.4 | -2.7 |
|  |  | % | -12.4 | -10.2 | -34.7 | -60.1 | -46.2 | -20.5 | 3.1 | 8.7 | 3.1 | 13.4 | 4.0 | 4.6 | -25.8 |
| **Central** |  |  |  |  |  |  |  |  |  |  |  |  |  |  |  |
| 2019 | 1,635,797 | Participant | 1,047,572 | 78,029 | 69,156 | 84,646 | 78,179 | 75,039 | 67,794 | 76,208 | 77,903 | 69,697 | 99,501 | 116,612 | 154,808 |
|  |  | Participants per 1,000 | 640 | 48 | 42 | 52 | 48 | 46 | 41 | 47 | 48 | 43 | 61 | 71 | 95 |
| 2020 | 1,600,580 | Participant | 932,888 | 64,569 | 48,598 | 30,757 | 72,686 | 61,976 | 77,690 | 84,405 | 77,087 | 75,442 | 101,169 | 121,987 | 116,522 |
|  |  | Participants per 1,000 | 583 | 40 | 30 | 19 | 45 | 39 | 49 | 53 | 48 | 47 | 63 | 76 | 73 |
|  |  | %p | -5.8 | -0.7 | -1.2 | -3.3 | -0.2 | -0.7 | 0.7 | 0.6 | 0.1 | 0.5 | 0.2 | 0.5 | -2.2 |
|  |  | % | -9.0 | -15.4 | -28.2 | -62.9 | -5.0 | -15.6 | 17.1 | 13.2 | 1.1 | 10.6 | 3.9 | 6.9 | -23.1 |
| **Southwestern** |  |  |  |  |  |  |  |  |  |  |  |  |  |  |  |
| 2019 | 1,378,301 | Participant | 898,762 | 66,274 | 63,967 | 73,567 | 66,291 | 63,045 | 58,098 | 65,683 | 63,676 | 57,747 | 85,137 | 99,827 | 135,450 |
|  |  | Participants per 1,000 | 652 | 48 | 46 | 53 | 48 | 46 | 42 | 48 | 46 | 42 | 62 | 72 | 98 |
| 2020 | 1,336,719 | Participant | 772,410 | 56,215 | 42,211 | 33,238 | 40,083 | 55,671 | 69,520 | 66,381 | 63,220 | 61,759 | 87,155 | 101,303 | 95,654 |
|  |  | Participants per 1,000 | 578 | 42 | 32 | 25 | 30 | 42 | 52 | 50 | 47 | 46 | 65 | 76 | 72 |
|  |  | %p | -7.4 | -0.6 | -1.5 | -2.9 | -1.8 | -0.4 | 1.0 | 0.2 | 0.1 | 0.4 | 0.3 | 0.3 | -2.7 |
|  |  | % | -11.4 | -12.5 | -32.0 | -53.4 | -37.7 | -8.9 | 23.4 | 4.2 | 2.4 | 10.3 | 5.6 | 4.6 | -27.2 |
| **Southeastern** |  |  |  |  |  |  |  |  |  |  |  |  |  |  |  |
| 2019 | 3,062,547 | Participant | 1,875,669 | 110,937 | 108,544 | 145,670 | 143,171 | 141,826 | 129,088 | 144,136 | 148,639 | 121,845 | 178,481 | 221,570 | 281,762 |
|  |  | Participants per 1,000 | 612 | 36 | 35 | 48 | 47 | 46 | 42 | 47 | 49 | 40 | 58 | 72 | 92 |
| 2020 | 3,011,588 | Participant | 1,645,210 | 96,071 | 79,722 | 46,696 | 75,088 | 116,095 | 146,852 | 162,315 | 151,316 | 138,051 | 184,610 | 229,138 | 219,256 |
|  |  | Participants per 1,000 | 546 | 32 | 26 | 16 | 25 | 39 | 49 | 54 | 50 | 46 | 61 | 76 | 73 |
|  |  | %p | -6.6 | -0.4 | -0.9 | -3.2 | -2.2 | -0.8 | 0.7 | 0.7 | 0.2 | 0.6 | 0.3 | 0.4 | -1.9 |
|  |  | % | -10.8 | -11.9 | -25.3 | -67.4 | -46.7 | -16.8 | 15.7 | 14.5 | 3.5 | 15.2 | 5.2 | 5.2 | -20.9 |
