# Supplementary material for: The impact of COVID-19 on screening for colorectal, gastric, breast, and cervical cancer in Korea
Source: Epidemiol Health. 2022 Jun 21;44:e2022053. doi: 10.4178/epih.e2022053 (PMC9754922; doi:10.4178/epih.e2022053)
Supplement: Supplementary Material 7. — Breast Cancer Screening Participation Rate Change (2019 vs. 2020, % change and % point difference) per Month by Geographical Region [file epih-44-e2022053-suppl7.docx]

Supplementary Material 7. Breast Cancer Screening Participation Rate Change (2019 vs. 2020, % change and % point difference) per Month by Geographical Region

|  | Eligible population |  | Total | Jan | Feb | Mar | Apr | May | Jun | Jul | Aug | Sep | Oct | Nov | Dec |
| --- | --- | --- | --- | --- | --- | --- | --- | --- | --- | --- | --- | --- | --- | --- | --- |
| **Total** |  |  |  |  |  |  |  |  |  |  |  |  |  |  |  |
| 2019 | 6,109,269 | Participant | 3,894,928 | 214,127 | 232,934 | 305,764 | 292,901 | 290,877 | 268,935 | 298,842 | 307,646 | 259,522 | 386,241 | 448,773 | 588,366 |
|  |  | Participants per 1,000 | 638 | 35 | 38 | 50 | 48 | 48 | 44 | 49 | 50 | 42 | 63 | 73 | 96 |
| 2020 | 5,912,890 | Participant | 3,299,516 | 177,016 | 148,205 | 109,132 | 161,413 | 239,075 | 294,452 | 319,079 | 306,096 | 284,091 | 405,357 | 454,590 | 401,010 |
|  |  | Participants per 1,000 | 558 | 30 | 25 | 18 | 27 | 40 | 50 | 54 | 52 | 48 | 69 | 77 | 68 |
|  |  | %p | -8.0 | -0.5 | -1.3 | -3.2 | -2.1 | -0.7 | 0.6 | 0.5 | 0.1 | 0.6 | 0.5 | 0.3 | -2.8 |
|  |  | % | -12.5 | -14.6 | -34.3 | -63.1 | -43.1 | -15.1 | 13.1 | 10.3 | 2.8 | 13.1 | 8.4 | 4.7 | -29.6 |
| **Capital** |  |  |  |  |  |  |  |  |  |  |  |  |  |  |  |
| 2019 | 2,917,160 | Participant | 1,859,299 | 78,947 | 97,715 | 136,161 | 135,250 | 137,865 | 131,366 | 142,281 | 149,522 | 128,041 | 193,708 | 228,151 | 300,292 |
|  |  | Participants per 1,000 | 637 | 27 | 33 | 47 | 46 | 47 | 45 | 49 | 51 | 44 | 66 | 78 | 103 |
| 2020 | 2,842,524 | Participant | 1,579,110 | 67,525 | 60,229 | 50,989 | 73,571 | 111,156 | 134,297 | 151,577 | 148,880 | 141,871 | 204,356 | 232,264 | 202,395 |
|  |  | Participants per 1,000 | 556 | 24 | 21 | 18 | 26 | 39 | 47 | 53 | 52 | 50 | 72 | 82 | 71 |
|  |  | %p | -8.2 | -0.3 | -1.2 | -2.9 | -2.0 | -0.8 | 0.2 | 0.5 | 0.1 | 0.6 | 0.5 | 0.4 | -3.2 |
|  |  | % | -12.8 | -12.2 | -36.7 | -61.6 | -44.2 | -17.3 | 4.9 | 9.3 | 2.2 | 13.7 | 8.3 | 4.5 | -30.8 |
| **Central** |  |  |  |  |  |  |  |  |  |  |  |  |  |  |  |
| 2019 | 845,308 | Participant | 550,477 | 40,280 | 37,757 | 47,073 | 42,277 | 40,928 | 36,240 | 41,480 | 42,036 | 36,230 | 52,253 | 57,301 | 76,622 |
|  |  | Participants per 1,000 | 651 | 48 | 45 | 56 | 50 | 48 | 43 | 49 | 50 | 43 | 62 | 68 | 91 |
| 2020 | 818,088 | Participant | 465,654 | 31,860 | 24,734 | 15,726 | 24,125 | 34,048 | 41,911 | 45,643 | 42,001 | 38,699 | 54,051 | 59,202 | 53,654 |
|  |  | Participants per 1,000 | 569 | 39 | 30 | 19 | 29 | 42 | 51 | 56 | 51 | 47 | 66 | 72 | 66 |
|  |  | %p | -8.2 | -0.9 | -1.4 | -3.6 | -2.1 | -0.7 | 0.8 | 0.7 | 0.2 | 0.4 | 0.4 | 0.5 | -2.5 |
|  |  | % | -12.6 | -18.3 | -32.3 | -65.5 | -41.0 | -14.0 | 19.5 | 13.7 | 3.2 | 10.4 | 6.9 | 6.8 | -27.6 |
| **Southwestern** |  |  |  |  |  |  |  |  |  |  |  |  |  |  |  |
| 2019 | 722,990 | Participant | 478,206 | 35,922 | 36,260 | 41,414 | 36,226 | 34,541 | 31,642 | 35,925 | 35,103 | 30,611 | 44,831 | 49,646 | 66,085 |
|  |  | Participants per 1,000 | 661 | 50 | 50 | 57 | 50 | 48 | 44 | 50 | 49 | 42 | 62 | 69 | 91 |
| 2020 | 692,605 | Participant | 399,709 | 29,341 | 22,001 | 17,120 | 21,857 | 30,588 | 37,633 | 34,414 | 33,960 | 31,995 | 47,299 | 49,624 | 43,877 |
|  |  | Participants per 1,000 | 577 | 42 | 32 | 25 | 32 | 44 | 54 | 50 | 49 | 46 | 68 | 72 | 63 |
|  |  | %p | -8.4 | -0.7 | -1.8 | -3.3 | -1.9 | -0.4 | 1.1 | 0.0 | 0.0 | 0.4 | 0.6 | 0.3 | -2.8 |
|  |  | % | -12.7 | -14.7 | -36.7 | -56.8 | -37.0 | -7.6 | 24.2 | 0.0 | 1.0 | 9.1 | 10.1 | 4.3 | -30.7 |
| **Southeastern** |  |  |  |  |  |  |  |  |  |  |  |  |  |  |  |
| 2019 | 1,623,811 | Participant | 1,006,946 | 58,978 | 61,202 | 81,116 | 79,148 | 77,543 | 69,687 | 79,156 | 80,985 | 64,640 | 95,449 | 113,675 | 145,367 |
|  |  | Participants per 1,000 | 620 | 36 | 38 | 50 | 49 | 48 | 43 | 49 | 50 | 40 | 59 | 70 | 90 |
| 2020 | 1,559,673 | Participant | 855,043 | 48,290 | 41,241 | 25,297 | 41,860 | 63,283 | 80,611 | 87,445 | 81,255 | 71,526 | 99,651 | 113,500 | 101,084 |
|  |  | Participants per 1,000 | 548 | 31 | 26 | 16 | 27 | 41 | 52 | 56 | 52 | 46 | 64 | 73 | 65 |
|  |  | %p | -7.2 | -0.5 | -1.1 | -3.4 | -2.2 | -0.7 | 0.9 | 0.7 | 0.2 | 0.6 | 0.5 | 0.3 | -2.5 |
|  |  | % | -11.6 | -14.8 | -29.8 | -67.5 | -44.9 | -15.0 | 20.4 | 15.0 | 4.5 | 15.2 | 8.7 | 4.0 | -27.6 |
