# Supplementary material for: The impact of COVID-19 on screening for colorectal, gastric, breast, and cervical cancer in Korea
Source: Epidemiol Health. 2022 Jun 21;44:e2022053. doi: 10.4178/epih.e2022053 (PMC9754922; doi:10.4178/epih.e2022053)
Supplement: Supplementary Material 8. — Cervical Cancer Screening Participation Rate Change (2019 vs. 2020, % change and % point difference) per Month by Geographical Region [file epih-44-e2022053-suppl8.docx]

Supplementary Material 8. Cervical Cancer Screening Participation Rate Change (2019 vs. 2020, % change and % point difference) per Month by Geographical Region

|  | Eligible population |  | Total | Jan | Feb | Mar | Apr | May | Jun | Jul | Aug | Sep | Oct | Nov | Dec |
| --- | --- | --- | --- | --- | --- | --- | --- | --- | --- | --- | --- | --- | --- | --- | --- |
| **Total** |  |  |  |  |  |  |  |  |  |  |  |  |  |  |  |
| 2019 | 8,299,528 | Participant | 4,799,842 | 259,335 | 293,668 | 371,375 | 361,461 | 362,170 | 334,993 | 374,057 | 386,381 | 330,360 | 458,267 | 532,491 | 735,284 |
|  |  | Participants per 1,000 | 578 | 31 | 35 | 45 | 44 | 44 | 40 | 45 | 47 | 40 | 55 | 64 | 89 |
| 2020 | 8,120,142 | Participant | 4,240,658 | 224,132 | 203,542 | 184,694 | 235,130 | 318,237 | 373,885 | 401,164 | 382,134 | 369,141 | 488,353 | 546,788 | 513,458 |
|  |  | Participants per 1,000 | 522 | 28 | 25 | 23 | 29 | 39 | 46 | 49 | 47 | 45 | 60 | 67 | 63 |
|  |  | %p | -5.6 | -0.4 | -1.0 | -2.2 | -1.5 | -0.4 | 0.6 | 0.4 | 0.1 | 0.6 | 0.5 | 0.3 | -2.5 |
|  |  | % | -9.7 | -11.7 | -29.2 | -49.2 | -33.5 | -10.2 | 14.1 | 9.6 | 1.1 | 14.2 | 8.9 | 5.0 | -28.6 |
| **Capital** |  |  |  |  |  |  |  |  |  |  |  |  |  |  |  |
| 2019 | 4,108,335 | Participant | 2,411,769 | 116,302 | 138,957 | 178,849 | 177,370 | 181,224 | 171,968 | 186,873 | 194,791 | 168,406 | 236,942 | 277,147 | 382,940 |
|  |  | Participants per 1,000 | 587 | 28 | 34 | 44 | 43 | 44 | 42 | 45 | 47 | 41 | 58 | 67 | 93 |
| 2020 | 4,053,003 | Participant | 2,139,826 | 103,702 | 95,940 | 94,095 | 116,492 | 157,506 | 181,778 | 201,556 | 193,152 | 190,148 | 252,867 | 286,979 | 265,611 |
|  |  | Participants per 1,000 | 528 | 26 | 24 | 23 | 29 | 39 | 45 | 50 | 48 | 47 | 62 | 71 | 66 |
|  |  | %p | -5.9 | -0.3 | -1.0 | -2.0 | -1.4 | -0.5 | 0.3 | 0.4 | 0.0 | 0.6 | 0.5 | 0.3 | -2.8 |
|  |  | % | -10.1 | -9.6 | -30.0 | -46.7 | -33.4 | -11.9 | 7.1 | 9.3 | 0.5 | 14.5 | 8.2 | 5.0 | -29.7 |
| **Central** |  |  |  |  |  |  |  |  |  |  |  |  |  |  |  |
| 2019 | 1,122,616 | Participant | 645,731 | 41,127 | 43,246 | 53,619 | 49,659 | 48,749 | 43,678 | 49,614 | 51,258 | 44,412 | 59,891 | 66,926 | 93,552 |
|  |  | Participants per 1,000 | 575 | 37 | 39 | 48 | 44 | 43 | 39 | 44 | 46 | 40 | 53 | 60 | 83 |
| 2020 | 1,094,473 | Participant | 569,714 | 33,933 | 30,557 | 25,123 | 32,780 | 43,648 | 50,512 | 54,009 | 50,718 | 48,515 | 63,705 | 69,446 | 66,768 |
|  |  | Participants per 1,000 | 521 | 31 | 28 | 23 | 30 | 40 | 46 | 49 | 46 | 44 | 58 | 63 | 61 |
|  |  | %p | -5.5 | -0.6 | -1.1 | -2.5 | -1.4 | -0.4 | 0.7 | 0.5 | 0.1 | 0.5 | 0.5 | 0.4 | -2.2 |
|  |  | % | -9.5 | -15.4 | -27.5 | -51.9 | -32.3 | -8.2 | 18.6 | 11.7 | 1.5 | 12.0 | 9.1 | 6.4 | -26.8 |
| **Southwestern** |  |  |  |  |  |  |  |  |  |  |  |  |  |  |  |
| 2019 | 930,931 | Participant | 527,705 | 30,992 | 35,216 | 43,233 | 39,625 | 38,682 | 35,125 | 41,340 | 41,946 | 36,291 | 50,416 | 56,115 | 78,724 |
|  |  | Participants per 1,000 | 567 | 33 | 38 | 46 | 43 | 42 | 38 | 44 | 45 | 39 | 54 | 60 | 85 |
| 2020 | 905,455 | Participant | 468,560 | 26,186 | 23,740 | 24,048 | 27,983 | 36,308 | 43,913 | 41,046 | 40,024 | 39,294 | 54,287 | 57,061 | 54,670 |
|  |  | Participants per 1,000 | 517 | 29 | 26 | 27 | 31 | 40 | 48 | 45 | 44 | 43 | 60 | 63 | 60 |
|  |  | %p | -4.9 | -0.4 | -1.2 | -2.0 | -1.2 | -0.1 | 1.1 | 0.1 | -0.1 | 0.4 | 0.6 | 0.3 | -2.4 |
|  |  | % | -8.7 | -13.1 | -30.7 | -42.8 | -27.4 | -3.5 | 28.5 | 2.1 | -1.9 | 11.3 | 10.7 | 4.5 | -28.6 |
| **Southeastern** |  |  |  |  |  |  |  |  |  |  |  |  |  |  |  |
| 2019 | 2,137,646 | Participant | 1,214,637 | 70,914 | 76,249 | 95,674 | 94,807 | 93,515 | 84,222 | 96,230 | 98,386 | 81,251 | 111,018 | 132,303 | 180,068 |
|  |  | Participants per 1,000 | 568 | 33 | 36 | 45 | 44 | 44 | 39 | 45 | 46 | 38 | 52 | 62 | 84 |
| 2020 | 2,067,211 | Participant | 1,062,558 | 60,311 | 53,305 | 41,428 | 57,875 | 80,775 | 97,682 | 104,553 | 98,240 | 91,184 | 117,494 | 133,302 | 126,409 |
|  |  | Participants per 1,000 | 514 | 29 | 26 | 20 | 28 | 39 | 47 | 51 | 48 | 44 | 57 | 64 | 61 |
|  |  | %p | -5.4 | -0.4 | -1.0 | -2.5 | -1.6 | -0.5 | 0.8 | 0.6 | 0.1 | 0.6 | 0.5 | 0.3 | -2.3 |
|  |  | % | -9.5 | -12.1 | -27.7 | -55.2 | -36.9 | -10.7 | 19.9 | 12.4 | 3.3 | 16.0 | 9.4 | 4.2 | -27.4 |
