# Supplementary material for: The impact of COVID-19 on screening for colorectal, gastric, breast, and cervical cancer in Korea
Source: Epidemiol Health. 2022 Jun 21;44:e2022053. doi: 10.4178/epih.e2022053 (PMC9754922; doi:10.4178/epih.e2022053)
Supplement: Supplementary Material 9. — Cancer Screening Participation Rate in 2019 and 2020 considering the 6-month extension of the NCSP for the eligible population of 2020 [file epih-44-e2022053-suppl9.docx]

Supplementary Material 9. Cancer Screening Participation Rate in 2019 and 2020 considering the 6-month extension of the NCSP for the eligible population of 2020

|  |  | Gastric | Breast | Cervical |
| --- | --- | --- | --- | --- |
| 2019 | Eligible Population | 11,625,627 | 6,109,269 | 8,299,528 |
|  | Participant | 7,194,489 | 3,894,928 | 4,799,842 |
|  | Participants  per 1,000 | 619 | 638 | 578 |
|  | Participation Rate (%) | 61.9 | 63.8 | 57.8 |
| 2020 | Eligible Population | 11,439,268 | 5,912,890 | 8,120,142 |
|  | Participant | 6,244,491 | 3,299,516 | 4,240,658 |
|  | Participants  per 1,000 | 546 | 558 | 522 |
|  | Participation Rate (%) | 54.6 | 55.8 | 52.2 |
|  | %p | -7.3 | -8.0 | -5.6 |
|  | % | -11.8 | -12.5 | -9.7 |
| 2021  (Jan-Jun) | Additional Participants | 525,404 | 261,872 | 302,472 |
|  | Participants per 1,000 | 46 | 44 | 37 |
|  | Additional Participation Rate (%) | 4.6 | 4.4 | 3.7 |
| 2020-2021 | Final Participants | 6,769,895 | 3,561,388 | 4,543,130 |
|  | Final Participants per 1,000 | 592 | 602 | 559 |
|  | Final Participation Rate (%) | 59.2 | 60.2 | 55.9 |
| 2019 vs.  2020-2021 | %p | -2.7 | -3.6 | -1.9 |
|  | % | -4.4 | -5.6 | -3.3 |
